# Historical Analyses of Disordered Handwriting: Perspectives on Early 20th-Century Material From a German Psychiatric Hospital

**DOI:** 10.1177/0741088316681988

**Published:** 2016-12-21

**Authors:** Markus Schiegg, Deborah Thorpe

**Affiliations:** 1 University of Augsburg, Lehrstuhl für Deutsche Sprachwissenschaft, Augsburg, Germany; 2 University of York, Heslington, York, UK

**Keywords:** handwriting, disorder, 20th century, psychiatry, neurology, Germany

## Abstract

Handwritten texts carry significant information, extending beyond the meaning of their words. Modern neurology, for example, benefits from the interpretation of the graphic features of writing and drawing for the diagnosis and monitoring of diseases and disorders. This article examines how handwriting analysis can be used, and has been used historically, as a methodological tool for the assessment of medical conditions and how this enhances our understanding of historical contexts of writing. We analyze handwritten material, writing tests and letters, from patients in an early 20th-century psychiatric hospital in southern Germany (Irsee/Kaufbeuren). In this institution, early psychiatrists assessed handwriting features, providing us novel insights into the earliest practices of psychiatric handwriting analysis, which can be connected to Berkenkotter’s research on medical admission records. We finally consider the degree to which historical handwriting bears semiotic potential to explain the psychological state and personality of a writer, and how future research in written communication should approach these sources.

On May 11, 1784, Ann Ferguson of Perth, Scotland, suffering from diverse ailments, wrote a letter to Dr. William Cullen, the owner of a successful private medical practice, with a request for advice. Ferguson’s writing is distinctive for its messy layout, with uneven inking and ill-formed letters that present the reader with problems deciphering the words easily. Thus, Cullen’s response begins, “I have with the utmost difficulty read your letter and if you do not hereafter write to me more distinctly or get some body to do it for you I will not look at any letter you write me.”^1^ Cullen’s statement reveals that he considered Ferguson able to regulate her handwriting quality consciously, and did not recognize any diagnostic potential in her disorderly writing. Despite Ferguson’s reports about her severe headaches on one side of her head, Cullen’s reply focused upon her piles as the presumed cause of her agony. When Ferguson wrote again to report the failure of the prescribed treatment, Cullen further disparaged her poor writing—“your writing is hardly more intelligible, therefore it gives me pain to advise on such imperfect accounts,” thereby reiterating his sole interest in the *contents* of her letter.

Though handwritten correspondence seems archaic today—a niche distinct from the prevalent modes of typed communication—there is, as the following sections of our article show, interest in handwriting from within writing and communication studies, especially relating to the potential benefits of handwriting for cognition, creativity, and idea generation. Handwriting also offers vital diagnostic potential in modern medicine. As the product of complex cognitive and motor abilities, writing provides insight into an array of medical conditions that affect these abilities. Thus, modern neurology utilizes writing and drawing tasks in the diagnosis and monitoring of diseases and disorders. However, there has hitherto been little consideration of how modern medical handwriting analysis can inform our knowledge of historical experiences of disorders that affect written communication. With this in mind, we first set out the methodological challenges of comparing the writing of modern individuals with confirmed medical diagnoses with script by historical individuals with *suspected* disorders.

Around a hundred years after Ann Ferguson wrote her letter to Dr. Cullen, we see evidence for a shift in medical perception of the relationship between handwriting and health. With the rise of psychiatry as a scientific discipline in the 19th century, psychiatrists developed empirical methods to assess patients’ speech and writing. Thus, our article moves on to describe the development of handwriting analysis in the history of psychiatry, and delineates its relationship with modern neurology. This alignment is useful because, though neuroscience was advancing rapidly by the close of the 19th century, the treatment and care of patients with neurological conditions was often performed in a psychiatric context.^2^

In our study, we examine unpublished handwritten material, writing tests and letters, from patients in an early 20th-century psychiatric hospital in southern Germany (Irsee/Kaufbeuren). We have chosen this material because it provides us novel insights into the earliest practices of psychiatric handwriting analysis and because we are able to present a hitherto underresearched historical text type with new opportunities for the examination of disordered handwriting. Our goal is to show through different case studies how the scrutiny of historical handwriting can recontextualize individual writing disorders, as well as enhance our understanding of historical contexts of writing. Our article outlines how novel interdisciplinary research is combining historical handwriting analysis with modern medicine to offer insight into medical conditions that involve writing disorders. After our empirical analyses, we finally evaluate the degree to which historical handwriting reflects the psychological state and personality of a writer, and we investigate how future researchers in written communication should approach these sources.

This article outlines historical evidence that handwriting analysis was, in the context of the Irsee/Kaufbeuren hospital, used by doctors to complement verbal accounts of a patient’s history. We demonstrate that both had considerable illocutionary force; they contributed to the documentation that Berkenkotter and Hanganu-Bresch have shown was “the legal/medical means through which an individual ceased to be an autonomous agent” and was committed to asylum confinement. Combined with the admissions records and case histories, medical handwriting examination represented “a certification of unsound mind” (Berkenkotter & Hanganu-Bresch, 2011, p. 221).

According to Austin’s (1962, p. 117) concept of “uptake,” the patient’s confinement was the “uptake”—that is, the real-world effect—of the written documentation, the “discursive work” produced around his or her admission (Berkenkotter & Hanganu-Bresch, 2011, p. 242). Handwriting analysis combined with the patient’s clinical case history produced a “strategic narrative” and “the primary channel for communicating information on clinical presentations” (Berkenkotter, 2008, p. 18). Our article shows that after the initial examination, ongoing analysis of a patient’s written communication—both content and form—contributed to the continued confinement of the individual.

## Handwriting in Writing Studies, Modern Neurology, and Early Psychiatry

### Handwriting in Writing Studies

Handwriting is gaining increasing attention within writing studies research. Van Dijck and Neef (2006, p. 7) explain that, in the age of hypermedia and the predominance of the digital communication, handwriting has been dismissed as “a backward technique, a slowly deteriorating and gradually vanishing tradition.” However, they argue that handwriting’s previous adaptability should ensure its ongoing longevity: “[it] has always adjusted its use and meaning in the face of larger technological, social, and cultural transformations” (Van Dijck & Neef, 2006, p. 8). The peculiar features—and potential advantages—of handwriting for planning, creativity, and idea generation have been well documented. By the 1980s, research had already discussed the different planning processes employed by writers using word processing from those using pen and paper (Haas, 1989). Recent research has demonstrated the value of note taking by hand for information retention and understanding (Mueller & Oppenheimer, 2014).

Research has identified four key faculties responsible for the writing process, whether employing pen and ink or finger and keyboard: (a) the working memory (“[the] capacity to hold varying amounts of information in memory while processing it”; MacArthur & Graham, 2016, p. 29), (b) transcription skills (“transcribing the words the writer wants to say into written symbols on the page,” which includes handwriting, keyboarding, and spelling; MacArthur & Graham, 2016, p. 31), (c) self-regulation (this includes planning and evaluation), and (d) motivation (e.g., the attitude toward writing and self-efficacy). The close interrelationship between these faculties has been demonstrated in the triangle model of the internal functional writing system (Berninger & Winn, 2006, p. 97). This model puts the working memory in the center of a triangle, leading the “cognitive flow” for text generation, transcription, and various elements of executive functioning such as planning, reviewing, revising, and self-monitoring.

Where there are disruptions to these writing processes, the underlying disorder could be one of a variety of different types. These broadly include neurodevelopmental disorders (such as dyslexia and handwriting disorders experienced in childhood; see Rosenblum, Weiss, & Parush, 2003), acquired disorders (those caused by brain damage, infection, or as a side effect of medication, for example), and interruptions to normal brain ageing caused by conditions such as Alzheimer’s disease and Parkinson’s disease, as we explain in more detail below. In addition, an individual’s writing may be affected by a combination of two or more different disorders. Developmental disorders in children and teenagers are attracting increasing research attention (see the research overview in James, Jao, & Berninger, 2016). However, the focus of this article is on the latter two types of handwriting distortions: those caused by acquired disorders and neurological and/or psychiatric conditions that present symptoms and progress in adulthood (especially later adulthood).

### Writing and Drawing Tasks in a Modern-Day Neurological Context

Where modern neurology is concerned with handwriting, it focuses on relationships between the different stages of the writing process, particularly “correlations between working memory and writing fluency and quality” (MacArthur & Graham, 2016, p. 29). Motor dysfunctions and cognitive impairment (especially visuospatial deficits) can be deduced from impaired transcription skills caused by neurological conditions (S. Wang, Bain, Aziz, & Liu, 2005, pp. 188-189). To identify such disorders, writing and drawing tasks are employed routinely in a clinical context. Digital graphic analysis can also be conducted to gauge the severity and progression of degenerative conditions such as Parkinson’s disease objectively. Researchers may use computerized tools such as a digitizing pen and tablet, collecting temporal information and data about pen pressure (i.e., how quickly, or rhythmically, the pen moves) in addition to spatial data (Caligiuri, Teulings, Dean, Niculescu, & Lohr, 2010).

Methods of clinical writing analysis can be split roughly between (a) those that assess the patient’s ability to create standardized shapes, such as a spiral-drawing task, and (b) handwriting analysis, that is, an evaluation of the qualities of written letters and words. (a) In a spiral-drawing task, a person is shown an Archimedean spiral (enlarging circles moving out from a central point), and asked to trace it (Bain, Findley, Atchison, et al., 1993). This noninvasive, easy-to-administer method is especially useful for characterizing tremor, the most common movement disorder (Alty & Kempster, 2011). Compared with writing words, drawing shapes requires the person to execute standard figures, and so extracts more comparable information (e.g., drawing shape and consistency; H. Wang et al., 2008, p. 264). The spiral shape is a suitable method of differentiating types of tremor because the task involves both the distal and proximal arm joints, and different types of tremor distort the controlled coordination of these joints in different ways (S. Wang et al., 2005, pp. 188-189). Though Parkinsonian and essential tremor can sometimes be difficult to separate using clinical examination, it is important to make this separation in order to determine the correct prognosis and treatment of the condition, and a spiral-drawing task can help (Hess & Pullman, 2012, p. 2; Bajaj, Knöbel, Gontu, Bain, 2011). For example, a small drawn spiral can be indicative of Parkinson’s disease, displaying the micrographia or small, cramped, drawing that is sometimes caused by the condition, and patients with ataxia will produce ill-formed spiral shapes (Alty & Kempster, 2011, p. 624).^3^

(b) Handwriting analysis—the study of the forms, layout, language, and content of handwritten letters and words—allows insight into the impact of disorders on cognitive functioning. Handwriting “entails an intricate blend of cognitive, kinesthetic, and perceptual-motor components”^4^ and—unlike drawing spirals—demands abilities in spelling, grammar, and punctuation (Rosenblum et al., 2003). As in the spiral analysis, researchers may take the handwriting sample on a digitizing tablet, and so gather quantitative data of the writing features (Rosenblum, Engel-Yeger, & Fogel, 2013). Writing tasks that assess pen pressure and velocity as well as spatial measures can be used to differentiate people with Parkinson’s disease from healthy controls (Rosenblum et al., 2013). Cognitive deficits in writing have been reviewed in relation to the major forms of dementia. Studies have found that in Alzheimer’s disease-type dementia there are many types of deficit in narrative writing, including the intrusion of incorrect or irrelevant information and perseveration on certain words and phrases (Graham, 2000). The use of digitizing tablets has revealed that the impact of dementia extends beyond the memory and planning processes involved in writing; it also affects motor programs. Yan, Rountree, Massman, Doody, and Li (2008) have examined the influence of Alzheimer’s disease and mild cognitive impairment on handwriting-like movement, finding that both conditions are associated with deterioration in fine motor control and coordination, especially evident in slowed handwriting performance. In addition, healthy controls performed handwriting-like movements more smoothly than individuals with these disorders, suggesting interference with the fluidity of movements.

Handwriting assessments have pushed into the hinterland between the disciplines of “neurology” and “psychiatry.” For example, Caligiuri et al. (2010) have used digitizing tablets to quantify the effects of antipsychotic medication on the motor system of patients with schizophrenia. Using sentence-writing tasks, they found abnormalities associated with certain medications in several kinematic features of handwriting, including increased movement duration (“bradykinesia”)—which is also a symptom of Parkinson’s disease. This study exemplifies the value of handwriting analysis in medicine; the authors conclude that the findings of the study indicate “that the measurement of handwriting kinematics is an objective behavioural biomarker of the effects of antipsychotic medication on the motor system” (Caligiuri et al., 2010, p. 81).

In addition to its use in a clinical context, handwriting analysis can be employed in forensics. Posthumous handwriting analysis has been used in cases of contested last wills. The handwriting of individuals with neurological conditions can be challenged as forgery due to its difference from a specimen signature, usually taken when the subject was not exhibiting signs of a movement disorder. However, close inspection has shown that handwriting changes due to disorder are distinguishable from the indicia of forgery (Walton, 1997). In addition to confirming identity, handwriting analysis has also been used to gauge the cognitive state of a writer in the case of a disputed will (Fontana, Dagnino, Cocito, & Balestrino, 2008).

### Handwriting Analysis in the Early Years of Psychiatry

The analysis of handwriting in a clinical context can be traced back to the early years of psychiatry.^5^ Over the course of the 19th century, psychiatry was transformed into a natural science and became institutionalized (Schott & Tölle, 2006, chap. 28). Psychiatrists such as Wilhelm Griesinger (1817–1868) established empirical methods for patient assessment and therapy. In addition to detailed examinations of the patient’s physical constitution, psychiatrists found it increasingly important to test a patient’s mental capacities, among them his or her ability to speak and write.

With Emil Kraepelin (1856–1926), psychiatry was established as a scientific discipline (Schott & Tölle, 2006, p. 123). In his highly influential textbook on psychiatry (Kraepelin, 1883), he refers frequently to patients’ writings in the diagnosis of their condition. He provides several transcriptions of excerpts from patient letters, diaries and other texts, which he analyzes in regard to form and content; in his 7th edition (1904) he reproduces 14 short facsimiles of handwriting that illustrate changes in handwriting, which he assigned to various illnesses. Kraepelin uses a scale to measure the pressure of writing in patients’ manic and depressive phases, a methodology that bears some similarity to the evaluation of pen pressure in modern neurological diagnostic context (p. 515). He also demonstrates handwriting changes during the progression of an illness, by comparing the handwriting of two patients with “Dementia paralytica” (pp. 308-309) and by showing differences in writing after a “paralytic attack” (p. 311). Other major publications proceeded similarly (see, e.g., Bleuer 1916).

The use of handwriting as a diagnostic tool was not restricted to German psychiatry. The French pathologist and neurologist Jean-Martin Charcot (1825–1893) discusses handwriting samples in his Parisian *Lectures on the Diseases of the Nervous System* (1879). In addition, in his book *Mad Humanity* (1898), the British psychiatrist Forbes Winslow (1844–1913) dedicates a whole chapter to “handwriting of the insane” (pp. 87-118). This evidence demonstrates that distinguished psychiatrists and neurologists of the late 19th and early 20th century used their patients’ handwriting as a diagnostic tool. Despite their interest in writing, however, these samples provided only one of many windows into their patients’ conditions. In addition, these doctors demonstrated awareness that some people presenting psychiatric symptoms were still able to write fluent letters (Kraepelin, 1904, p. 409; Sommer, 1901, p. 105; Winslow, 1898, p. 87).

We now move on to focus on the role of handwriting analysis in a particular geographical context: the Irsee/Kaufbeuren Psychiatric Hospital in Bavaria, Germany. We first discuss the methodological challenges in historical handwriting analysis from a modern research perspective. Then, we proceed to examine the role of handwriting analysis at this hospital in the early 20th century, providing a selection of case studies of patients who were under examination.

## Disordered Written Communication and Early 20th-Century Patients of the Irsee/Kaufbeuren Psychiatric Hospital

### Methodological Challenges in Historical Handwriting Analysis

Early psychiatrists benefitted from direct access to their patients; they could investigate the broader contexts of their lives and examine their illnesses from a variety of angles—as do modern neurologists when they conduct patient assessments.^6^ In contrast, when we study historical communication, we work with the limited data that have survived to the present day. In this research, we are historicizing handwriting in a clinical context and the further back we work, the more difficult this task becomes. The difficulties of this historical work are compounded by the problems inherent in examining handwriting, in isolation, for insight into medical disorders.

Document analyst Roy A. Huber has expressed caution about the use of handwriting as a diagnostic tool, arguing that there is no reliable evidence that changes in writing can occur before the other symptoms of an illness. While handwriting deterioration is an “accepted consequence of many kinds of illness,” Huber contends that “there are few characteristics of this deterioration that may be identified with any particular illness, or family of illnesses” (Huber & Headrick, 1999, p. 200). However, in contrast with forensic document examiners, humanities scholars are not expected to prove claims “beyond reasonable doubt” in a way that will stand up in court (Haeton, 2005). So, in analyzing this historical handwriting, we make suggestions that—in the absence of more convincing alternatives and based on the weight of the evidence—can be accepted with caution.

Where neurologists have studied historical material, they have not used handwriting in isolation as a diagnostic tool. Their research, hitherto focusing on the handwriting of socially privileged writers from the 17th to the 20th centuries, has benefitted from information about the person’s lifestyle and the symptoms of the condition from their diaries and letters. For example, Louis and Kavanagh (2005) found evidence for essential tremor in the writing of John Adams (1735–1826), one of the signers of the American Declaration of Independence. Kempster and Alty (2008) have examined the art critic John Ruskin’s (1819–1900) relationship between illness and the form and content of writing. Finally, Williams, Holmes, Kemper, and Marquis (2003) conducted a comparative analysis of 57 letters written by King James VI/I (1566–1625), where they correlated the king’s decline in written language complexity with historical reports of his illness.

In contrast, scholars of earlier handwriting, and writing by less privileged people, often work with limited biographical information. This is problematic, since information about family history provides important insight into conditions that are understood to be hereditary. In addition, handwriting may be affected by a confusing intermingling of concurrent diseases and disorders. For example, an individual with Parkinson’s disease could also have arthritis, which would have its own impact on their movement abilities. Finally, we must recognize that handwriting is influenced by learned writing styles, environmental conditions, and writing technologies (such as stylus type, and paper/parchment quality). Thus, it is important that we investigate the cultural context of historical handwritten sources, which presents complexities that make a straightforward comparison with modern writing unhelpful.

Despite the problems inherent in studying handwriting for insight into historical disorders, a small number of studies have reached convincing conclusions from linguistic and paleographical features alone. Research has demonstrated that it *is* possible to distinguish graphetic and linguistic features in writing that suggest one type of disorder or another. Herkenrath (1987) and Petersohn (1996) found linguistic evidence for the writing deficits suggesting dyslexia in the work of 12th- and 13th-century German scribes writing in Latin. These results are of relevance to both philology and the medical humanities; they suggest further reasons for textual variants, to be taken into consideration when editing a text; in addition, they demonstrate very early cases of dyslexia, lending support to the argument that the disorder is a universal human disposition (Petersohn, 1996, p. 589). More recently, Thorpe and Alty’s (2015) study of the handwriting of the anonymous 13th-century monk at Worcester Cathedral Priory known as the “Tremulous Hand of Worcester” found that the fine amplitude, regular tremor visible in the handwriting suggested essential tremor rather than any other kind of condition. The lack of cognitive difficulties experienced by this scribe, together with the lack of small handwriting (“micrographia”), helped to rule out Parkinson’s disease.

This article proceeds to demonstrate how an interdisciplinary approach to disordered handwriting can enhance the study of disordered written communication. It takes a paleographical approach to writing samples from the Irsee/Kaufbeuren hospital, combined with linguistic analysis, to shed more light upon the writing difficulties experienced by these historical individuals. Several aspects of the material chosen for this article are unprecedented and will broaden our understanding of historical written communication. First, in examining previously unpublished early 20th-century patient records from a psychiatric hospital, we present novel insights into the earliest practices of psychiatric handwriting analysis. Second, we analyze a hitherto underresearched historical text type—letters written by patients from psychiatric hospitals—with new opportunities for the examination of disordered handwriting. Third, the case studies demonstrate how the scrutiny of historical handwriting can recontextualize individual writing disorders, as well as enhance our understanding of historical contexts of writing.

### Context

In an early 19th-century attempt to improve the condition of mental health care in Bavarian Swabia (west Bavaria), the former Benedictine monastery in Irsee in the rural Allgäu area was transformed into a psychiatric hospital (Schmidt, Kuhlmann, & v. Cranach, 2012). It opened in 1849 under the director Friedrich W. Hagen, a follower of the growing scientific perspective on psychiatry. The hospital’s good reputation prompted a large extension in nearby Kaufbeuren, which opened in 1876; this institution still exists today as a psychiatric hospital (Bezirkskrankenhaus Kaufbeuren). The hospital archive stores tens of thousands of patient files from 1849 onward, together with the former doctors’ library, which contains a worn copy of the 7th edition (1904) of Kraepelin’s fundamental work on psychiatry. This volume allows us to look over the shoulders of the hospital’s doctors; many of its passages are underlined with pencil, among them remarks on speech disorders caused by “dementia paralytica” (pp. 306 and 390). Also present is the 2nd edition of Bleuer’s textbook (1918), which includes sections on disordered handwriting. The existence of these texts in the library suggests that the Irsee/Kaufbeuren doctors kept in touch with the latest psychiatric methods of speech and handwriting analysis.

But how far was handwriting analysis used in the day-to-day operation of the hospital? Scrutiny of historical patient files from this institution reveals the degree to which doctors applied these methods in their everyday practice. They suggest that the doctors did perceive speech and handwriting analysis as important tools for diagnosis. These documents demonstrate that patients were either asked to provide short samples of their script, or their handwriting was collected from more natural contexts of text production, for example, when they wrote a letter to a friend or family member.^7^ With this in mind, we first focus on samples taken during the initial examination, before moving on to consider how doctors analyzed patient correspondence.

### Handwriting Analysis During the Initial Examination

In the initial examination, the Irsee/Kaufbeuren doctors assessed new patients’ life history, their family health history, and their physical and psychiatric constitution. To assess the latter, patients were given various cognitive tests for their general knowledge, their competence at short calculations, and their ability to understand and produce both spoken and written words. The patients’ files summarize the results of these tests and give an initial diagnosis, which formed the foundation for the chosen therapy. The 19th-century files, especially, often do not provide systematic documentation of the initial examination. In this period, this examination appears to have differed in quality and quantity according to the psychiatrist responsible. The 20th-century notes became more extensive, occasionally including small slips of paper containing writing samples by the patients. These sheets were inserted into the report, with the doctors’ handwritten comments next to them.

**Figure 1.**
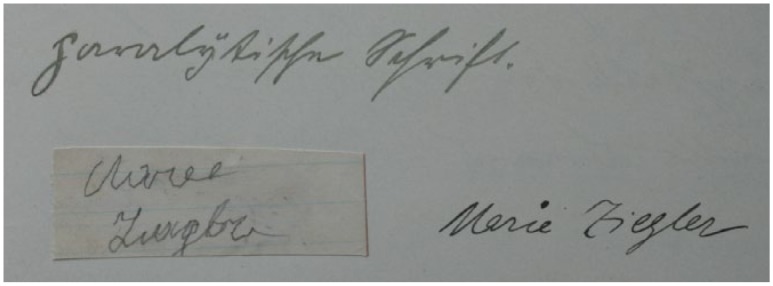
Writing test by Marie Ziegler, bottom left (year of admission: 1912). Archiv des Bezirkskrankenhauses Kaufbeuren, file 1760. Note: File numbers refer to the patient files belonging to the Bezirkskrankenhaus Kaufbeuren, Germany, and are deposited in their archive.

In Figure 1, we see a record of the initial examination of Marie Ziegler, which took place in 1912. This patient was 39 years old with progressive para-lysis, a late-stage symptom of syphilis. Doctors undertook a comprehensive analysis of her language competence and analyzed both speech and writing. She was asked to write down her name on a small slip, which was glued into the report, along with the doctor’s description of it as “paralytic script.” Further notes from this initial examination reveal that Ziegler presented “severe speech dysfunction” because of her “childish, quirky way of speaking” and her “hesitations, stumbling syllables, sometimes stammering simultaneously.”

According to Kraepelin (1904, p. 308), the main characteristics of a “paralytical script” are irregular and unstable characters. Ziegler’s letter forms in Figure 1 do show minor distortions; though the initial *M* and *Z* are executed confidently, the subsequent letters are less well defined in comparison with the doctor’s writing to the right of the sample. This is especially apparent in Ziegler’s conjoined *g* and *l*, which show distinctive signs of shakiness. We must recognize that this is a signature—a mode of writing that is sometimes stylized and often rushed, and therefore usually not representative of the writer’s usual script. However, an examination of other documents by Ziegler in her file, namely three letters and two scrap papers full of her writing, reveals more severe distortions.

**Figure 2.**
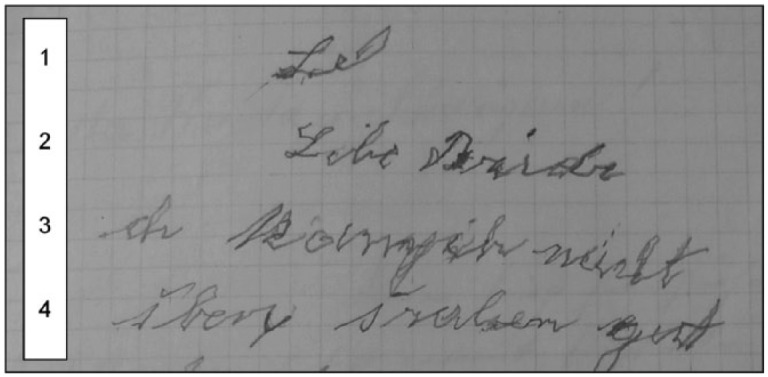
Beginning of a letter by Marie Ziegler (June 1912). Archiv des Bezirkskrankenhauses Kaufbeuren, file 1760.

Figure 2 shows the beginning of a letter written by Ziegler three weeks after her admission. In line 1, she begins the word *Liebe* (“Dear”), but interrupts the formation of the *b*. She starts again in the next line, writing *Libe Bridr* (“Dear brothers”). The spelling is phonetic, that is, she omits the letter *e* twice. The first omission is in *Li* ***e****be*, an *e* that is not pronounced and usually indicates a long vowel, and the second is in the final syllable of *Brid* ***e****r*. The use of *i* in *Brider*, instead of standard German *Brüder*, indicates an unrounded vowel, which is a common feature in informal and spoken German. Using these spoken forms in writing is not in itself a symptom of disorder—in fact, it is commonly observed in texts written by people with less formal education (Schiegg, 2015). However, the irregular and trembling shape of Ziegler’s letter forms do hint at a disorder. Lines 3 and 4 are barely legible and further idiosyncratic spellings such as *šraben* probably for *schreiben* (“write”; second word in line 4) appear, as well as corrections (see the ending of the second word in line 3). These distortions considered, the features of this letter support the doctors’ judgment that there was unusual instability in Maria Ziegler’s handwriting.

**Figure 3.**
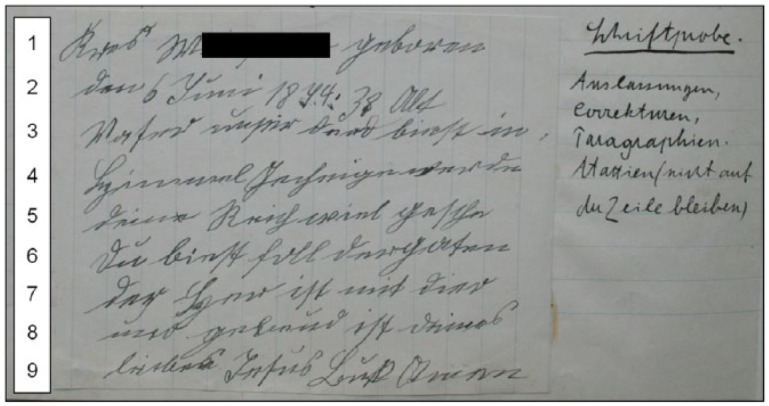
Writing test by Kreszenz W. (left), with doctor’s notes (right). Year of admission: 1912. Archiv des Bezirkskrankenhauses Kaufbeuren, file 1878. Full name has been redacted by Schiegg/Thorpe.

In Figure 3, we see the initial examination of another patient, who was also classified as suffering from paralysis, the 39-year-old Kreszenz W.^8^ The doctors first analyzed the patient’s spoken language and her problems with reading a text aloud (doctor’s note: “drops single letters, syllables, words, phrases”). Then the patient wrote a short text, comprising nine lines, on a piece of paper: her name, birthday and (parts of) the Lord’s Prayer and the Hail Mary. A doctor commented on this “writing test” and stated: “omissions, corrections, *Paragraphien, Ataxien* (not keeping in the line [see note 3])” (see Figure 3, annotation to right).

A close examination of this patient’s writing sample reveals the extent of her linguistic omissions. First, her rendering of the two prayers features clipped and intermingled sentences. However, this may have been conducted intentionally by the writer, to fit the text on the small sheet of paper. Further small omissions are made, such as one letter in line 3 (*der d*[*u*] *biest*; “which art”) and a syllable in the last word of line 5 (*gesche*[*he*]; “will be done”). Such shortenings may again be an interference with spoken language. However, the extent of the spelling and writing irregularities in this text suggests a pathological cause. There are multiple corrections, including in line 5 (*e* is crossed out) and in line 9 (an *n* is changed into an *s*). *Paragraphien* can be defined as confusions of words and letters, and the appearance of an *s* instead of a *t* in *Vaser* (line 3; “father”) could be interpreted as an example of this (Pschyrembel, 2014, p. 1597). Some erroneous words are similar to the intended ones (line 4: *Gecheige* for *Geheiligt* “hallowed” and line 8 *gebeud* for *gebenedeit* “blessed”), which suggests a deficit in the language domain of cognition. Ataxia, a loss of coordination (see note 3) may be observed—as the doctor explains—in the patient’s difficulty in keeping the line. This feature is more obvious in this patient’s letters than in the writing test, probably because the latter involved a degree of self-consciousness under examination conditions.

We now proceed to consider, in more detail, the value of these patient letters of correspondence, both for the historical physicians of Irsee/Kaufbeuren and for scholars of written communication today.

### Handwriting Analysis of Patient Letters of Correspondence at Irsee/Kaufbeuren

According to the Irsee/Kaufbeuren statutes, all letters written by or to patients had to be examined by the director, and they were sometimes not forwarded to their intended recipients (Schiegg, 2015). There are two letters written by Kreszenz W. and kept in her file that, like Marie Z.’s letters, confirm the observations made from her writing test. They present several omissions and corrections as well as features of spoken language. Notes made by the Irsee/Kaufbeuren doctors demonstrate that such letters were a useful source of information about patient health. In the patients’ files, we often find doctors’ comments written directly onto their letters or into their medical histories. For example, we find the following note in Kreszenz W.’s medical history: “Has written a letter to her husband that abounds with omissions, *Paragraphien* etc.” Physicians saw a clear connection between a patient’s deteriorating health and a decline in writing abilities. Such a link can, for example, be observed for Balbina H. (no. 1996) in whose file we find two letters. The later one is, according to the doctors, in “very poor shape,” “both in regards to form and content,” which “hints at the increasing deterioration of her mental activities” (Schiegg, 2015, p. 83).

**Figure 4.**
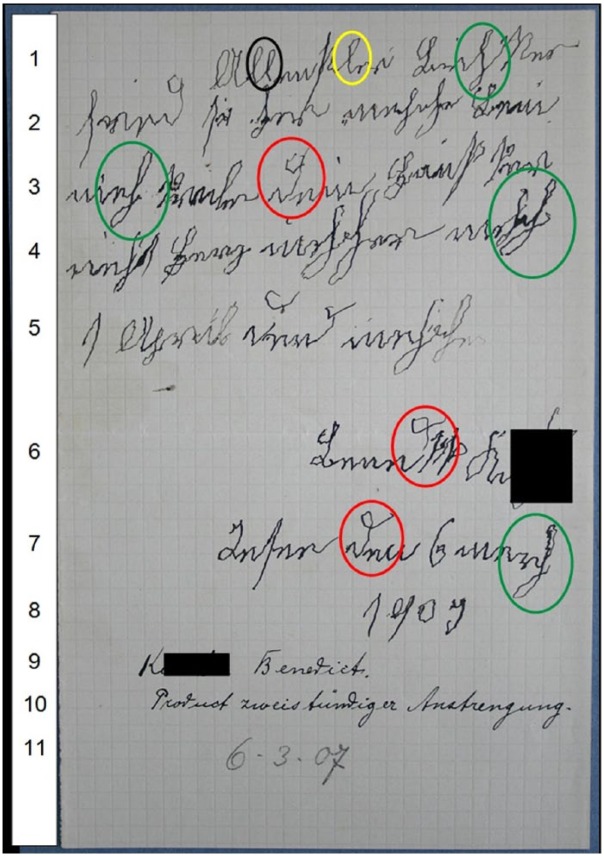
Letter by Benedikt K. (March 1907); Archiv des Bezirkskrankenhauses Kaufbeuren, file 1676. The circled *l* and *ll* on line 1 demonstrate the variability of tremor severity between individual letter forms. The circled letters *d* (lines 3, 6, and 7) and *h* (lines 1, 3, 4, and 7) show the multidirectional feature of the tremor, impacting both vertical and horizontal strokes, and affecting the full extent of letter loops.

Moving on to consider a further case study from Irsee/Kaufbeuren, day laborer Benedikt K., was diagnosed with “dementia senilis” and was 67 years old when he was admitted to the hospital in September 1906.^9^ Figure 4 shows a letter by him, displaying severely distorted handwriting; this letter was kept by the doctors in his file.

In classifying different types of abnormal movement, Kraepelin (1904, p. 479) associated a “high regularity and extensity of the individual movements” with “senile tremor.” The letters in Figure 4 are indeed unusually large—the paper size is about A5, with only 8 lines of writing. This enlarged writing is consistent with studies demonstrating significant differences between age groups in the spatial domain of handwriting even in healthy individuals, with increases in letter size typically occurring with age (Rosenblum et al., 2013). However, contrary to Kraepelin’s statement, the tremor is not “regular”; there is little regularity in the extent to which individual letter shapes are distorted by tremor. For instance, the double *l* in the first word of Figure 4 shows very little evidence of tremor, whereas the last *l* in this word exhibits a prominent tremor. In addition, the tremor intrudes into letter strokes in various directions; it is not only present in the downward strokes, but also appears in horizontal sections of letters. See for example the shapes of his *d* in lines 3, 6, and 7. This is particularly evident in *h*, a letter that has two loops and reaches both below the base line and above the mean line. In this letter, we see distortions caused by tremor in every part of the letter, extending around the entirety of both loops (lines 1, 3, 4, and 7). This contrasts with the medieval “Tremulous Hand of Worcester,” in whose handwriting we see a tremor that is considerably more prominent in vertical sections of letters (Thorpe & Alty, 2015). Variability in the pressure being exerted upon the page is a dominant feature in line 5 of this letter. The second letter *h* of the last word in this line—it is not possible to make sense of this word—is notably smaller and fainter with the first, suggesting the increasing difficulty of the writing task for this patient as he struggled with his motor control.

This difficulty is confirmed by a doctor’s note at the foot of the page (lines 9-11), in which he stated the patient’s name, commented that the letter was a “product of two hours’ effort,” and provided the date. This short description of the writing process is remarkable; it offers us information about Benedikt K’s slow writing speed and the considerable effort he had to put into this piece of work. For skilled adult writers, the transcription process is “mostly an unconscious, automatic task” (MacArthur & Graham, 2016, p. 31). In contrast, extended writing task performance time is associated with both extremes of the human lifespan. It has been shown that for beginner writers the transcription task requires effort and attention (Berninger, 1999). Studies of elderly writers have reported an increase in time spent with the pen in air rather than on paper, especially in the age group 60 to 94, suggesting less automatism in writing performance with age (Rosenblum et al., 2013). Patients considered not to be “healthy”—for example, those with mild cognitive impairments and mild Alzheimer’s disease—spent significantly longer time with the pen in air than “healthy” participants (Werner, Rosenblum, Bar-On, Heinik, & Korczyn, 2006). Benedikt K’s disorder evidently compromised his working memory, and thus the dynamic features of writing. It appears that his condition affected his text generation and transcription skills as well as his general ability to plan and execute activities in relation to space and time (Berninger & Winn, 2006, p. 97; Rosenblum et al., 2013).

The fact that Benedikt K. was determined to write despite experiencing difficulties with the task demonstrates the importance that he attributed to this letter. Although many of the words are illegible or lack coherence, the text can clearly be identified as correspondence. It has each of the structural components of a letter: an indented address line (line 1), his signature (line 6), as well as the place (“Irsee”) and date of writing (lines 7-8). He dates the letter to 1909, although it was actually written in 1907 (see the doctor’s annotation). This may be a sign of his temporal disorientation, but it could also be a simple mistake, as the day and month are correct (“6 March”). The letter is evidently a private one, as the address pronoun in singular shows (*dein*; line 3). Benedikt K. mentions “your house” (*dein Haus*; line 3) and the word “heart” (*Herz*; line 4). Analogous to the contents of several other patient letters, this could be interpreted as a wish to be accommodated in the house of the addressee, whom he knows personally. He also mentions *1 April* (line 5), which may be the date that he anticipates his wish could be performed—about four weeks from the point of writing. So, despite its handwriting distortions and linguistic irregularities, Benedikt K.’s text can be considered a functioning letter. This intimate and informal piece of correspondence, which assumes prior knowledge from the correspondent, would likely have been better understood by its intended addressee than by modern scholars.

## Discussion

On November 5, 1994, Ronald Reagan distributed an autograph letter, which informed the public about his diagnosis with a neurodegenerative disorder: “My Fellow Americans, I have recently been told that I am one of the millions of Americans who will be afflicted with Alzheimer’s disease” (cited from Chen, 2012, p. 10). This two-page letter has “uneven margins, [an] unsteady ductus, and hand-blackened errors” (Chen, 2012, p. 10). It evoked compelling responses. Edmund Morris (1995) wrote in *The* *New Yorker*: “I confess that I, too, cried at that letter, with its crabbed script and enormous margin (so evocative of the blizzard whitening his mind).” These emotional reactions were provoked less by the message itself than by its form of presentation—from the embodiment of the writer in his handwritten text. According to Bolter and Gruisin’s (1999) theory of “remediation,” Reagan’s handwriting can be described as “hypermediated”: “the viewer is meant to notice and interpret the medium as carrying a message of its own” (Chen, 2012, p. 19).

Our article has demonstrated that disordered handwriting of individuals from the Irsee/Kaufbeuren hospital carries its own semiotic potential. Despite the methodological challenges that we face when analyzing historical handwriting from a modern perspective, we have demonstrated that the scrutiny of historical handwriting can recontextualize individual writing disorders, as well as significantly enhance our understanding of historical contexts of writing and of the individual writers themselves. Our examination of how handwriting was used in the early years of psychiatry has presented novel insights into the earliest practices of psychiatric handwriting analysis that can be aligned with its application in modern neurology as part of the diagnosis and disambiguation of a range of conditions. Letters written by patients from psychiatric hospitals are a hitherto underresearched historical text type that allows new opportunities for the examination of disordered handwriting. Thus, we are able to show that an awareness of pathological aspects in handwriting can be relevant to our understanding of written communication.

The focus of our article was on evidence for interruptions in the cognitive and motor processes of individuals with disorders. But to what, if any, extent can handwriting reveal the psychological state of its writer—his or her “ways of thinking”? The British Institute of Graphologists advertises that “handwriting reveals how the writer thinks, feels and behaves, and it does so directly and immediately. It shows the motivation that lies behind actions, and outlines the writer’s propensity to behave in ways that may not be expected” (cf. British Institute of Graphologists, n.d.). Graphology has been, and still is, used in the assessment of prospective employees and partners, and even in assessing child development (Schofield, 2013). However, it is controversial and is not widely respected in modern scholarship (cf. Furnham, Chamorro-Premuzic, & Callahan, 2003). Though research has shown abnormalities in motor performance—and thus in handwriting—caused by psychological conditions such as major depressive disorder (Lohr, May, & Caligiuri, 2013), generalizations about personality based upon subjective visual analysis of handwriting features are to be considered pseudoscience (Goodwin, 2010, p. 36).

The data from the Irsee/Kaufbeuren hospital are well-suited for the analysis of historical handwriting distortions, because samples are often accompanied by patient histories and the evaluative notes of contemporary physicians. Moving forward, we propose that patient letters from this and other institutions can offer new insights into age-related handwriting and language changes, as well distortions resulting from psychiatric and/or neurological conditions. This material could, for example, provide a novel source to introduce a historical dimension to patholinguistics (Schiegg, 2015).^10^ Recent, interdisciplinary collaboration has demonstrated that even medieval handwriting—distanced from modern neurologists both temporally and culturally—contains recognizable signs of the impact of neurological diseases and disorders on handwritten communication (Thorpe & Alty, 2015). The ongoing challenge is to develop techniques for isolating and analyzing the most subtle effects of disorders in handwriting, both historical and modern.

Such endeavors may succeed only if we focus on source material using a synthesis of disciplinary approaches, particularly if we work with centuries-old material with limited contextual information. We need to combine medical knowledge with socio-historical and linguistic expertise; and meticulous philological study with paleographical examinations. Cognitive models of the writing process from writing studies should be joined with modern, medical methods of disordered handwriting analysis. Thereby, we will not only shed new light on the handwritten source material under investigation, but may simultaneously interrogate disciplinary boundaries, and thus reshape the constituent disciplines themselves.

## Notes

1. Taken from the Cullen Project: The Consultation Letters of Dr William Cullen (1710–1790) at the Royal College of Physicians of Edinburgh. This AHCR project at the School of Critical Studies (University of Glasgow) and the Royal College of Physicians of Edinburgh includes facsimiles and editions of Cullen’s archive of several thousand letters both from patients of close family members and Cullen’s responses. The correspondence between Cullen and Ann Ferguson (case ID 1677; 4 letters) can be consulted here: http://cullenproject.ac.uk/

2. For instance, Alois Alzheimer made his first observations of what would be termed Alzheimer’s disease in a psychiatric institute; it was not until after World War II that a practical division between neurology and psychiatry was made (Martin, 2002). To add to this disciplinary blurring, it is often from psychiatric institutes that the most significant historical handwriting data survive, often rich in evidence for tremor conditions and cognitive impairment.

3. Ataxia describes a group of conditions that affect coordination, balance, and speech, caused by damage to the cerebellum or other parts of the nervous system. In the writing context, it affects the balance of the fingers (Pschyrembel, 2014, p. 193).

4. In contrast to *kinematic*, which refers to the movement itself, *kinesthetic* has cognitive implications, implying “learning through movement” (Rosenblum, Weiss, & Parush, 2003).

5. For more on the theories on handwriting and psychiatric health proposed by Kraepelin and others, see recently Schäfer (2016).

6. Where we have quoted from source material in German from the Irsee/Kaufbeuren hospital, we have translated silently into English, providing analysis of the German language where we deemed it relevant to this research.

7. Both types of data collection appeared in the contemporary psychiatric literature; Charcot (1879) seems to have preferred short samples of name, date, and/or a sentence, while Kraepelin (1904) and Bleuer (1916) focused on excerpts or full letters written by patients.

8. This patient’s full surname has been redacted here in the interests of data protection.

9. As with the patient mentioned in note 8, this patient’s full surname has been redacted.

10. Wiley’s *Handbook of Language and Speech Disorders* (Damico, Müller, & Ball, 2010), for example, solely focuses on today’s language, speech, cognitive, and intellectual disorders. See similarly de Gruyter’s handbook on *Linguistic Disorders and Pathologies* (Blanken, Dittmann, Grimm, Marshall, & Wallesch, 1993).
